# Long-Term Follow up of Blinatumomab in Older Patients with B-Cell Acute Lymphoblastic Leukemia

**DOI:** 10.3390/ph17030335

**Published:** 2024-03-05

**Authors:** Yamini K. Kathari, Max An, Christine Dougherty, Ashkan Emadi

**Affiliations:** 1 Departments of Medicine, University of Maryland School of Medicine, Baltimore, MD 21201, USA; 2 Marlene and Stewart Greenebaum Comprehensive Cancer Center, Baltimore, MD 21201, USA; 3 Department of Pharmacology, University of Maryland School of Medicine, Baltimore, MD 21201, USA

**Keywords:** blinatumomab, older adult, Philadelphia negative B-cell ALL

## Abstract

Older adults who are diagnosed with acute lymphoblastic leukemia (ALL) and are treated with chemotherapy generally have poor outcomes. Blinatumomab is a CD19/CD3 bispecific T-cell engager that has been approved for the treatment of B-cell ALL in the relapsed/refractory setting or in patients with minimal residual disease (MRD) positivity. We previously reported on a small cohort of older adults with newly diagnosed Philadelphia chromosome negative B-cell ALL who were treated with blinatumomab monotherapy in the first line setting. This is a long-term follow up of those patients and their clinical courses. All five patients achieved complete remission (CR) after one cycle of blinatumomab, and three were MRD-negative. Two patients completed three cycles of blinatumomab, two patients completed four cycles of blinatumomab, and one patient completed 17 cycles of blinatumomab total. In the last four years, four of these patients had relapsed disease requiring additional therapy. Two patients are alive after 61 months and 57 months since their first cycle of blinatumomab. Two of the patients died at 10 months and one died at 20 months. Here we describe the long-term clinical courses of these patients.

## 1. Introduction

Patients of advanced age who are diagnosed with B-cell acute lymphoblastic leukemia (ALL) generally have worse prognosis and outcomes compared with younger patients, particularly adolescents and young adults (AYA). Older patients have medical comorbidities and may have lower functional status that may preclude them from receiving chemotherapy. These patients have higher early mortality, higher rates of relapse, and lower overall survival (OS), as compared to younger patients with ALL [1]. The five year OS is about 20% in patients over the age of 60 years who are diagnosed with B-cell ALL [2]. In contrast, AYA patients have a five year OS reaching between 80% and 85% [2,3]. These differences in outcomes may be attributed to the inability to tolerate intensive chemotherapy, and poor risk features of their ALL which may be related to advanced age (such as adverse-risk karyotype) [1,4,5]. Tyrosine kinase inhibitor (TKI) therapy can be used in patients with Philadelphia chromosome positive B-cell ALL, and these oral agents may be tolerated and have efficacy in the older population. However, TKIs are not used in Philadelphia chromosome negative B-cell ALL, leaving few therapeutic options for these patients. Moreover, older patients are often excluded from clinical trials, reducing other possible treatment choices. Therefore, using targeted agents or immunotherapy for older patients may be a more viable and tolerable approach. 

Blinatumomab is a CD19/CD3 bispecific T-cell engager that is currently approved by the United States Food and Drug Administration (FDA) for CD19 positive B-cell ALL in first or second complete remission (CR) with measurable (or minimal) residual disease (MRD) positivity or in relapsed or refractory B-cell ALL. We previously reported on a small cohort of five patients at the University of Maryland Greenebaum Comprehensive Cancer Center who were treated with frontline blinatumomab, showing that it was well tolerated and effective in treating B-cell ALL as a first line therapy [6]. Here, we present a long-term (four years) follow up of their clinical courses. 

## 2. Detailed Case Descriptions

**Figure 1:**
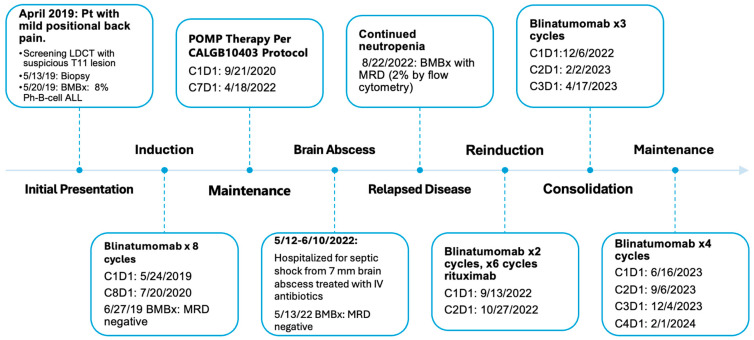
Clinical course and treatment for patient #4. BMBx—bone marrow aspiration and biopsy, LDCT—low dose CT scan.

This long-term follow up report contains five patients (Table 1), all of whom were the patients in the initial report [6]. Their ages ranged from 71 years to 86 years at the time of diagnosis. Two patients (patient #2 and patient #5) had therapy-related B-cell ALL as they previously were diagnosed with and treated for multiple myeloma or prostate cancer. Other medical comorbidities included, but were not limited to, hypertension, congestive heart failure (CHF), coronary artery disease (CAD), atrial fibrillation and dementia. All five patients in the cohort achieved CR after one cycle of blinatumomab monotherapy (given as a 28 day infusion) as the first line therapy. CR was defined as less than 5% lymphoblasts in bone marrow and no evidence of extramedullary disease elsewhere, red blood cell transfusion independent, with an absolute neutrophil count (ANC) greater than or equal to 1000/µL and platelets greater than or equal to 100,000/µL in the peripheral blood. These patients were also treated with standard-of-care prophylactic intrathecal chemotherapy with methotrexate, cytarabine (Ara C, cytosine arabinoside) and hydrocortisone once per cycle. Two patients (patient #1 and patient #2) completed three cycles of blinatumomab, two patients (patient #3 and patient #5) completed four cycles of blinatumomab, and one patient (patient #4) completed 17 cycles of blinatumomab total (eight cycles as first line therapy, and nine more cycles after relapsed disease, Figure 1). Four patients (#1, #2, #4, #5) experienced relapsed disease. Two patients (patient #3 and patient #4) are alive after 61 months and 57 months since their first cycle of blinatumomab. Two of the patients (#1 and #2) died approximately 10 months after diagnosis and one (patient #5) died at 20 months. 

Two patients remain alive at the time of this report approximately four years after the initial diagnosis of B-cell ALL. The first (patient #3) received a total of four cycles of blinatumomab and remained in MRD negative CR. This patient went on to receive maintenance POMP-like chemotherapy (oral 6-mecaptopurine, oral dexamethasone, oral methotrexate and intravenous vincristine) for 2 years, and currently remains in CR and off therapy. They are currently alive at 61 months since the diagnosis of B-cell ALL. The second patient (#4) received eight cycles of blinatumomab as first line therapy, which was followed by seven cycles of POMP-like maintenance per CALGB 10403 protocol [7]. While receiving maintenance therapy, they had a prolonged hospitalization (during which time their chemotherapy was held), where they were found to have brain abscesses requiring intravenous antibiotics with symptomatic and radiographic improvement. This patient was then found to have relapsed disease 15 months after their initial B-cell ALL diagnosis with mild to moderate pancytopenia. Bone marrow aspiration and biopsy at that time showed MRD positive disease. The work up for pancytopenia around that time showed an elevated rheumatoid factor, and mildly positive ANA (titer of 1:80). Myeloperoxidase antibodies, ANCA and C3/C4 complements were all within normal limits. They were evaluated for Chimeric antigen receptor (CAR) T-cell therapy, but it was ultimately deemed that there was little benefit of this treatment. Given the robust response in the first line setting, they were restarted on blinatumomab with the addition of rituximab (given once every four weeks for six cycles). This patient achieved a CR after the first cycle of blinatumomab and received eight additional cycles of blinatumomab as consolidation and maintenance, per package insert recommendation. Following this, they continued to be actively surveilled for any evidence of recurrence of the disease. Repeat testing of his immunologic markers 20 months later showed negative results for all of the following tests: ANA, rheumatoid factor, myeloperoxidase antibodies, and ANCA, along with normal C3/C4 complement studies. This patient is currently alive at 57 months since the diagnosis of B-cell ALL. 

Two patients were found to have relapsed disease after the third cycle of blinatumomab. The first patient (patient #1) had evidence of extramedullary disease with lymphadenopathy on the positron emission tomography-computed tomography scan (PET/CT). They were started on inotuzumab ozogamicin, a CD22 antibody-drug conjugate, and received four cycles of therapy. Follow up imaging showed near-complete resolution of lymphadenopathy. However, they again relapsed with evidence of the disease progression on CT imaging with chloromas, malignant omental deposits, and subcutaneous abdominal nodules. After the development of malignant ascites and spontaneous bacterial peritonitis, along with functional status decline and prolonged hospitalization, the patient was transitioned to home hospice. The second patient (patient #2) was found to have relapsed B-cell ALL on bone marrow aspirate and biopsy. They were also treated with inotuzumab as a second line agent and received five cycles. Cycle six was delayed due to hepatotoxicity, during which time the patient was found to have another relapse. After being hospitalized for worsening leukocytosis, altered mental status, pneumonia, and subdural hematoma after several falls, they were transitioned to hospice. Both of these patients died approximately 10 months after their initial diagnoses of B-cell ALL. 

One patient (#5) was lost to follow up after the third cycle of blinatumomab. On representation, there was evidence of relapsed disease, and they were admitted for cycle four of blinatumomab. This course was complicated by an episode of grade 4 neurotoxicity, with the symptoms of an altered mental status, confusion, and a change in handwriting. These symptoms improved with the initiation of dexamethasone, and they were re-challenged with blinatumomab. However, they again developed symptoms of neurotoxicity with a change in mental status and handwriting. Due to this, blinatumomab therapy was discontinued and the patient was subsequently treated with four cycles of inotuzumab and achieved CR after the first cycle of inotuzumab. However, after a new diagnosis of cirrhosis and an inability to receive further treatments, they eventually transitioned to hospice, and died 20 months after the initial diagnosis of B-cell ALL.

Regarding safety and tolerability, in our cohort of patients, all five had evidence of neurotoxicity of any grade during their treatment with blinatumomab, requiring treatment with steroids. Patients #2, #3 and #4 had evidence of cytokine release syndrome (CRS), ranging from grade 2 to grade 3. Patient #4 developed a brain abscess during their clinical course, requiring intravenous antibiotics, with symptomatic and radiographic improvement following antimicrobial therapy. In the literature, a majority of patients (85–90%) who are treated with blinatumomab had grade 3 or higher adverse events [4,5].

We also investigated the initial presence of CD19 and its plasticity during the treatment and after the occurrence of relapse. The lymphoblasts in patient #1 were positive for CD19 at diagnosis; the presence of CD19 at relapse was unknown since the ALL relapsed with evidence of malignant ascites and extramedullary spread. For patient #2, CD19 was positive at diagnosis and remained positive (30%) at relapse. Patient #3’s ALL expressed CD19 at diagnosis and the patient has not relapsed. For patient #4, CD19 was positive at diagnosis, and the patient received eight cycles of blinatumomab, followed by evidence of relapse per MRD assessment with only 2% blasts on bone marrow aspirate measured using flow cytometry. Due to a low disease burden, the evaluation of CD19 at relapse was not possible, and the patient was retreated with blinatumomab for nine more cycles. They are currently alive and live independently. CD19 was expressed by ALL cells in patient #5 both at diagnosis and at relapse (100%).

## 3. Discussion and Conclusions

This small cohort of five older adults received blinatumomab in the first line setting for B-cell ALL. They tolerated the treatments well and had a good response to therapy. Prior to treatment, the Eastern Cooperative Oncology Group (ECOG) Performance Status for patients ranged from 0 to 2; one patient was ECOG 0, two patients were ECOG 1, and two patients were ECOG 2. All five patients experienced adverse effects such as neurotoxicity requiring steroids. Additionally, three patients had evidence of grade 2 to grade 3 CRS. While two of these patients had early relapse after four months, two patients relapsed after 10 or 39 months, and one patient did not experience any relapsed disease. Two of the patients received POMP-like maintenance therapy per the SWOG 1318 Phase II trial and had the best outcomes of this cohort of patients. 

This long-term follow up illustrates that older adults with newly diagnosed B-cell ALL can tolerate blinatumomab therapy and can have durable responses without significant treatment interruptions. Interestingly, patient #4 in this cohort was retreated with blinatumomab combined with rituximab after they were found to have MRD-positive relapsed disease. They again achieved CR with the re-initiation of blinatumomab, demonstrating that patients who have had a long-term response may benefit from a trial of blinatumomab, as monotherapy or in combination with other agents such as rituximab, when there is relapsed disease. This allows for further lines of treatment (such as inotuzumab) to be available as other options for treatment in the future. As a note, the decision for adding rituximab to the blinatumomab was made to enhance (or potentially synergize) the activity of both agents in case of low effector-to-target cell ratios and at low-antibody concentration based on pre-clinical [8] or clinical [9,10] reports.

CD19 is a 95 kDa transmembrane protein restricted to B-lineage cells and follicular dendritic cells. It is encoded by the *CD19* gene on the short arm of chromosome 16 (16p11.2). CD19 is frequently expressed on B-lymphoblasts at the time of diagnosis of B-cell ALL. The presence and intensity of CD19 can be altered after treatment with CD19-targeted immunotherapies [11]. There are several reported mechanisms for the disappearance of CD19 at the time of relapse, including convergence of acquired mutations and alternative splicing of CD19 [12], loss of heterozygosity in *CD19* gene at the time of CD19-negative relapse [13], retention of misfolded protein in the endoplasmic reticulum [8], and loss of CD81 expression which is required for CD19 trafficking [14]. All five patients reported here had CD19 expression at diagnosis, ranging from 71% to 99%. Four of the patients had relapsed disease, although patient #1 presented with extramedullary disease at the time of relapse and CD19 status at relapse is unknown for this patient. Upon relapse, patient #2 had 30% CD19 expression and patient #5 had 100% CD19 expression. However, patient #4 relapsed with only MRD positive disease with 2% blasts in the bone marrow aspirate using flow cytometry, making the accurate estimation of the expression of CD19 difficult.

There are relatively few clinical trials that enroll older patients over the age of 65 years, limiting treatment options for patients and data to drive clinical decision making in this patient population. Recently, the SWOG 1318 Phase II trial of blinatumomab followed by POMP maintenance in this older population showed 19 out of 29 patients achieved CR. The median age in this trial was 75 years, and the three year disease-free survival (DFS) was 37% and OS was 37% [15]. 

In conclusion, this study shows that blinatumomab was safely administered in elderly patients with newly diagnosed B-cell ALL. It is well tolerated and can produce durable complete remission in older patients with newly diagnosed B-cell ALL. The toxicities that the patients experienced during therapy were treated and they tolerated therapy well. Therefore, blinatumomab can be considered as a viable option for first line therapy in elderly patients who are not fit for more intensive chemotherapy regimens. Our study showed that all patients experienced complete remission after one cycle, and one patient remains in CR after over five years of follow up. Another patient had an excellent and durable response for 39 months after first line blinatumomab, and responded well to blinatumomab again when they were noted to have MRD positive disease relapse. Further research is needed to continue to explore other options for treatment in the elderly population that are tolerable, safe and efficacious.

## 4. Methods

We conducted a retrospective review of five patients from our previous study of older adults over the age of 70 years with newly diagnosed B-cell ALL who received blinatumomab as first line therapy at the University of Maryland Greenebaum Comprehensive Cancer Center, outside of a clinical trial. MRD negative was defined as the absence of abnormal lymphoblasts using flow cytometry performed by Hematologics, Inc. (Seattle, WA, USA), with the estimated lower level of detection of 0.02%. Relapse was defined as greater than or equal to 5% lymphoblasts in the bone marrow or peripheral blood, or evidence of extramedullary ALL. The duration of response was defined using the interval from CR to relapse or death from any cause. The OS was defined as the interval from ALL diagnosis to death from any cause. The date of last follow up was 8 February 2024. A waiver of Health Insurance Portability and Accountability Act (HIPAA) authorization for the release of the Protected Health Information (PHI) identified in the research application was reviewed and approved for this study by the University of Maryland Baltimore (UMB) Institutional Review Board (IRB). The study was conducted in accordance with the Declaration of Helsinki. The protocol was approved by the University of Maryland School of Medicine IRB (Project identification code: GCCC19138).

## Figures and Tables

**Table 1:** Patient characteristics and comorbidities.

	Patient 1	Patient 2	Patient 3	Patient 4	Patient 5
**Age at diagnosis**	86	76	75	71	76
**Gender**	Female	Female	Male	Male	Male
**Comorbidities**	Atrial fibrillation s/p ablation, MI, CHF	Multiple Myeloma, Dementia	Atrial fibrillation	Glaucoma, HTN	Prostate Cancer, CHF, CAD s/p MI and PCI, gout
**ECOG at diagnosis**	2	2	1	0	1
**Blinatumomab cycles**	3	3	4	8	4
**Maintenance therapy**	N/A	N/A	POMP maintenance × 2 years	POMP maintenance × 2 years	N/A
**Duration of response**	4 months	4 months	61+ months	39 months	10 months
**Second line therapy**	Inotuzumab	Inotuzumab	N/A	Blinatumomab + rituximab	Inotuzumab
**Number of cycles of 2nd line therapy**	4	5	N/A	9 cycles of blinatumomab, 6 cycles of rituximab	4
**Complications**	Malignant ascites, spontaneous bacterial peritonitis	Altered mental status, leukocytosis, subdural hematoma	N/A	Brain abscess	Cirrhosis
**Overall Survival**	~10 months	~10 months	61+ months	57+ months	20 months

CAD—coronary artery disease, CHF—congestive heart failure, HTN—hypertension, MI—myocardial infarction, PCI—percutaneous coronary intervention, POMP—prednisone, vincristine, methotrexate, 6-mercaptopurine, s/p—status post.

## Data Availability

Data are contained within the article.
